# A ruthenium(II) complex as turn-on Cu(II) luminescent sensor based on oxidative cyclization mechanism and its application in vivo

**DOI:** 10.1038/srep08172

**Published:** 2015-02-02

**Authors:** Yunfei Zhang, Zonglun Liu, Kui Yang, Yi Zhang, Yongqian Xu, Hongjuan Li, Chaoxia Wang, Aiping Lu, Shiguo Sun

**Affiliations:** 1 College of Science, Northwest A&F University, Yangling, Shaanxi, 712100, China; 2 College of Plant Protection, Northwest A&F University, Yangling, Shaanxi, China; 3 Key Laboratory of Eco-Textile, Ministry of Education, School of Textile and Clothing, Jiangnan University, 1800 Lihu Avenue Wuxi, 214122, China; 4 School of Chinese Medicine, Hong Kong Baptist University, Kowloon Tong, Hong Kong, China

## Abstract

Copper ions play a vital role in a variety of fundamental physiological processes not only in human beings and plants, but also for extensive insects and microorganisms. In this paper, a novel water-soluble ruthenium(II) complex as a turn-on copper(II) ions luminescent sensor based on *o*-(phenylazo)aniline was designed and synthesized. The azo group would undergo a specific oxidative cyclization reaction with copper(II) ions and turn into high luminescent benzotriazole, triggering significant luminescent increasements which were linear to the concentrations of copper(II) ions. The sensor distinguished by its high sensitivity (over 80-fold luminescent switch-on response), good selectivity (the changes of the emission intensity in the presence of other metal ions or amino acids were negligible) and low detection limit (4.42 nM) in water. Moreover, the copper(II) luminescent sensor exhibited good photostability under light irradiation. Furthermore, the applicability of the proposed sensor in biological samples assay was also studied and imaged copper(II) ions in living pea aphids successfully.

As an essential transition metal ion not only for human beings and plants but also for extensive insects and microorganisms, Cu(II) plays a vital role in a variety of fundamental physiological processes including neurotransmission, energy generation, iron transportation, pigmentation and scavenging of free radicals^1^,^2^. Moreover, the internal concentrations of Cu^2+^ in normal organisms are tightly regulated and disruption of the copper homeostasis often cause disease states or pathophysiological conditions^3^,^4^. For humans, alterations in the cooper homeostasis are connected to some serious neurodegenerative diseases^5^,^6^,^7^,^8^ and may cause gastrointestinal disturbance or damages to liver and kidney^9^. While, for insects such as aphids, excess or deficiency in Cu(II) not only hinders their normal growth and development but also affects their plant responses^10^,^11^,^12^,^13^, which is closely related to the damage extent with their host plants. In addition, due to their widespread use in industry and agriculture, cupric ions are also considered to be a significant environmental pollutant^14^. Consequently, developing robust and versatile methods to investigate the biological and environmental roles of copper(II) ions have been attracted extensive attentions.

Among the reported methods for copper ions detection, luminescent probes are extensively employed owing to their distinct advantages in sensitivity and biological imaging^15^. However, due to the intrinsic fluorescence quenching property of Cu^2+^ stemming from its paramagnetic nature, most hitherto reported Cu^2+^ sensors have shown a “turn-off” response via an electron/energy transfer process^16^,^17^,^18^. Although some luminescence “off-on” Cu^2+^ sensors with high selectivity^19^,^20^, nanomolar sensitivity^19^,^20^,^21^,^22^, good water solubility^20^,^23^, excellent photostability^24^ and long emission wavelength^25^,^26^ have been reported, sensors combining all these features are rare up to now^15^. Furthermore, the probes for Cu^2+^ detection in biological systems, such as different cancer cells^25^,^27^, rat hippocampal slices^25^,^27^, zebrafish^25^,^28^, human tissues^27^,^29^, herb leaves^30^ have been investigated, however, copper(II) imaging in insects is rarely reported. Definitely, developing new Cu^2+^-selective turn-on luminescent probes with excellent performance for diverse biological systems is still of importance and necessity.

Ruthenium(II) complexes are one type of potential candidates for environmental and biological Cu^2+^ probing, due to their good water solubility, high chemical and photostability, intense polarized luminescence, red emission, large Stokes shifts, and long lifetimes^31^. To date, some ruthenium(II) complex based luminescent probes for Cu^2+^ have been developed^24^,^32^,^33^,^34^,^35^,^36^,^37^,^38^,^39^,^40^,^41^. Unfortunately, as far as we know, there is only one example of luminescence enhancement Cu^2+^ sensor based on a ruthenium(II) complex (Rubp-Ptz) reported by Gopidas's group, which could detect micromolar amounts of Cu^2+^ in acetonitrile solution^24^. Herein, in this paper, we focus on the development of a turn-on ruthenium(II) complex based luminescent sensor with superior performance for Cu^2+^ detection and imaging.

Lee et al. reported a fluorescence turn-on chemodosimeter for Cu^2+^ based on oxidative cyclization of a non-emissive azoaniline into a highly fluorescent benzotriazole product, which can detect μM-level concentrations of Cu^2+^ in water at room temperature with the green emission^23^. Given the relatively high detection limit of the reported sensor, here, we designed and synthesized a novel ruthenium(II) complex RuMAZO (Fig. 1) with *o*-(phenylazo) aniline group as a turn-on luminescent sensor for Cu^2+^. The non-emissive RuMAZO in presence of copper(II) ions undergoes oxidative cyclization to form a highly luminescent product RuTAZO (Fig. 1).

## Results

**Figure 1:**
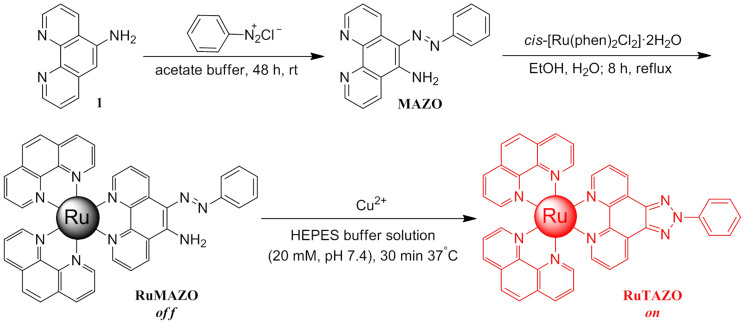
Synthesis of RuMAZO and the proposed mechanism of response of RuMAZO to Cu^2+^ ions.

The non-emissive RuMAZO in presence of copper(II) ions undergoes oxidative cyclization to form a highly luminescent product RuTAZO (Fig. 1). This cyclization reaction can be triggered by nM-level (4.42 nM) concentrations of Cu^2+^ in a HEPES (HEPES = 4-(2-hydroxyethyl)-1-piperazineethanesulfonic acid) buffer, to exhibit > 80-fold enhancement in a red emission at λ_max_em = 599 nm. The chloride salt of RuTAZO exhibits λ_max_em value at 599 nm with a quantum yield of 4.7%, using [Ru(bpy)_3_]^2+^ (bpy = 2,2-bipyridine) as a standard (see supplementary information part)^42^. Moreover, this probe has proved to be an appropriate luminescent Cu^2+^ imaging reagent in live pea aphis. To the best of our knowledge, this is the first report on developing a ruthenium(II) complex-based luminescent sensor for luminescence enhancement detecting Cu^2+^ in aqueous solution with high selectivity and sensitivity and imaging Cu^2+^ in insects.

**Figure 2:**
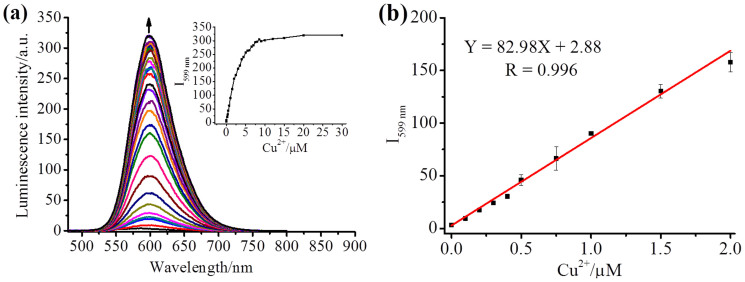
(a) Luminescence intensity of RuMAZO (10 μM) with various concentrations of Cu^2+^ (0–30 μM) in a HEPES buffer solution (20 mM, pH 7.4); Insert: the changes of luminescence intensity at 599 nm with various concentrations of Cu^2+^; (b) A linear correlation between emission intensity of RuMAZO at 599 nm and concentrations of Cu^2+^ (0.1–2.0 μM).

Luminescence enhancement was clearly evident up to 30 min in a HEPES buffer solution (20 mM, pH 7.4, 37°C), then no further significant changes occurred, indicating that the optimal reaction time for Cu^2+^ detection via oxidative cyclization for this sensor is around 30 min (Fig. S1). In addition, the luminescence properties of RuMAZO were checked under the same conditions. As shown in Fig. S2, after treatment with different concentrations (0–3 equiv.) of Cu^2+^ at a physiological temperature 37°C, the ligand absorption of RuMAZO (10 μM) at around 263 nm apparently increased and the metal-to-ligand charge transfer (MLCT) absorption at around 452 nm decreased, whereas a new ligand absorption peak at about 294 nm appeared. Correspondingly, within 30 min of reaction under the same conditions, the emission intensity at 599 nm increased to over 80 folds upon excitation at 465 nm with only 1 equiv. of Cu^2+^ (Fig. 2a). The Stokes shift of RuTAZO is 134 nm. These results indicate that the *o*-(phenylazo)aniline group of RuMAZO can be efficiently converted into luminescent benzotriazole. Furthermore, the dose-dependent luminescence enhancement followed a good linear relationship with very low Cu^2+^ concentrations in the range of 0.1–2.0 μM (Fig. 2b) and the limit of detection (LOD) for Cu^2+^ with RuMAZO (10 μM) was determined to be 4.42 × 10^−9^ M (see supplementary information part), lower or comparable to those of most previously reported highly sensitive sensors^15^. Thus, the broad linear range and low detection limit make RuMAZO suitable for environmental or biological copper(II) detection and imaging.

For further biological applications, the cytotoxicity of RuMAZO and Cu^2+^ to the HeLa cell lines was investigated with an MTT (3-(4,5-dimethylthiazol-2-yl)-2,5-diphenyltetrazolium bromide) assay after a 24 h treatment (Fig. S10). RuMAZO did not exhibit obvious cytotoxicity towards the HeLa cell lines at the concentrations employed. Confirming RuMAZO can be a suitable luminescence chemosensing probe for Cu^2+^ detection in vivo.

To investigate the practical applicability of RuMAZO as a Cu^2+^ sensor in the luminescence imaging of living cells, HeLa cells were incubated with RuMAZO (10 μM) for 2 h at 37°C in a PBS (phosphate buffer solution, pH = 7.4). After washed with PBS to remove the remaining RuMAZO, no obvious luminescence could be observed from the confocal laser scanning microscopy (Fig. S11a). However, the intracellular luminescence showed a clear red luminescence after incubated with Cu^2+^ (20 μM) and PDTC (pyrrolidine dithiocarbamate, 100 μM) for 2 h at 37°C (Fig. S11b). PDTC^43^ was used to increase the intracellular level of Cu^2+^. The results revealed that RuMAZO could be used as an off-on luminescent probe for imaging Cu^2+^ in living cells.

**Figure 3:**
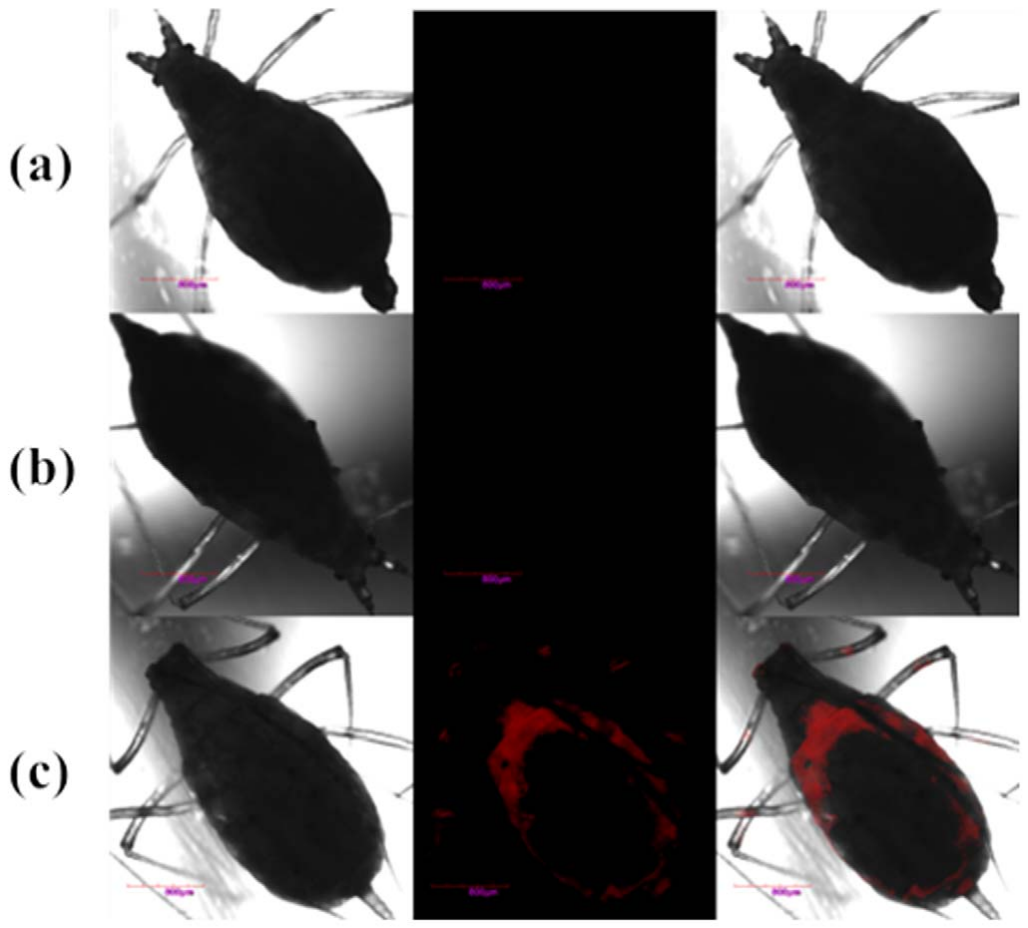
Confocal luminescence images of pea aphids given a subcutaneous injection of Cu^2+^ (a, 300 nL, 5 mM in a HEPES buffer solution (20 mM, pH 7.4)), 25 μM RuMAZO (b, 300 nL, 20 mM HEPES), 25 μM RuMAZO and 50 μM Cu^2+^ (c, 300 nL, 20 mM HEPES). Images were taken after incubation for 6 h. Left: Bright field images. Middle: Dark field images. Rigth: Merged images. λex = 488 nm.

To examine the applicability of the sensor for visualizing Cu^2+^ in living organisms, four-day-old pea aphids were selected and divided into three groups. The first two groups were given skin-pop injections at the bottom of the middle legs with Cu^2+^ (300 nL, 5 mM in a HEPES buffer solution (20 mM, pH 7.4)) or RuMAZO (300 nL, 25 μM in a HEPES buffer solution (20 mM, pH 7.4)) respectively as the control. The third group was given a hypodermic injection of 25 μM RuMAZO and then 50 μM Cu^2+^ (300 nL, 20 mM HEPES) immediately. All samples were imaged using a Confocal Laser Scanning Microscope with a 488 nm excitation laser after incubation for 6 h. As shown in Fig. 3, pea aphids in the experimental group exhibited distinct luminescence signal over the entire bodies. While no apparent emission was observed in the control groups, illustrating that RuMAZO could detect Cu^2+^ in vivo without the interference of background signals. Taken together, RuMAZO is proved to be a desired turn on imaging agent for visualizing the distribution of Cu^2+^ in insects, based on this, a reliable method could be established for investigating the functions of Cu^2+^ on the plant response of aphids, the work is ongoing now.

## Discussion

To investigate the sensing mechanism of RuMAZO to Cu^2+^, the reaction product of RuMAZO with Cu^2+^ in ethanol/H_2_O mixture was isolated and characterized by ^1^H NMR, ^13^C NMR and HR-MS (Fig. S19–S21). Furthermore, the isolated product exhibited nearly identical UV-vis and luminescence spectra with those of the testing mixture of RuMAZO and Cu^2+^ incubated at 37°C for 30 min (Fig. S3). The result of the EDTA (EDTA = ethylene diamine tetraacetic acid) competitive experiment provided further evidence on the non-binding interaction between RuMAZO and Cu^2+^ (Fig. S4). All these demonstrated the above mentioned proposed mechanism.

**Figure 4:**
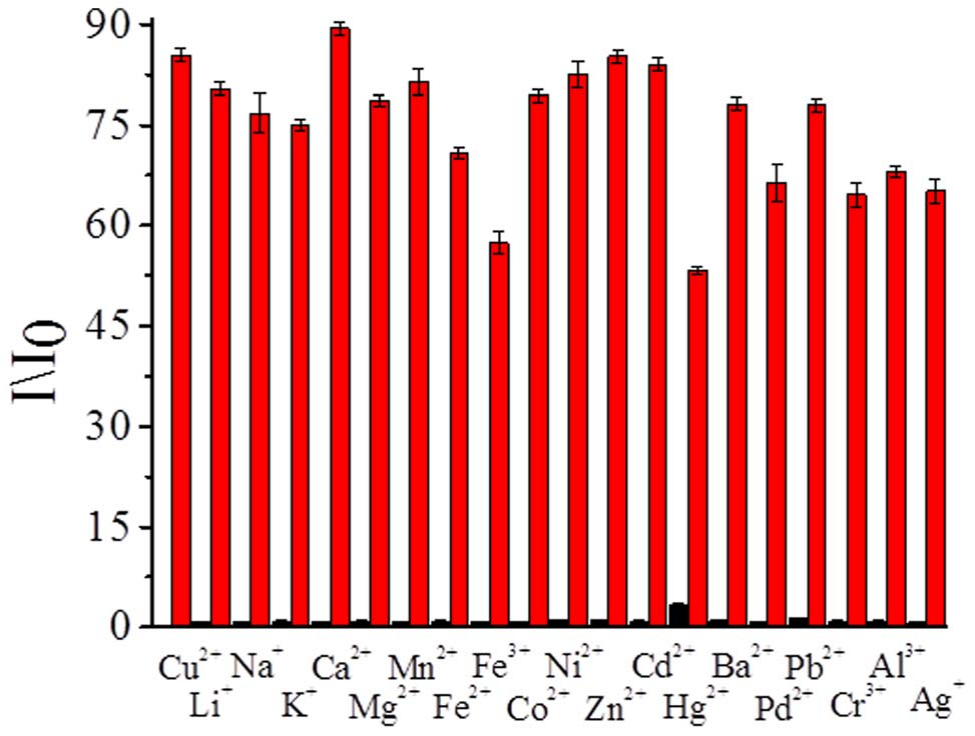
Luminescence changes of RuMAZO (10 μM) upon the addition of various metal ions (100 μM) and 10 μM Cu^2+^. Left-hand bars represent the luminescence response towards metal ions (blank, Li^+^, Na^+^, K^+^, Ca^2+^, Mg^2+^, Mn^2+^, Fe^2+^, Fe^3+^, Co^2+^, Ni^2+^, Zn^2+^, Cd^2+^, Hg^2+^, Ba^2+^, Pd^2+^, Pb^2+^, Cr^3+^, Al^3+^, Ag^+^); right-hand bars represent the subsequent addition of 10 μM Cu^2+^ to the aforementioned solutions.

To verify the selectivity of RuMAZO towards Cu^2+^, the influence of other metal ions on the sensing of Cu^2+^ was determined. As shown in Fig. 4 and Fig. S5, the changes of the emission intensity of RuMAZO in the presence of 10.0 equivalents of other metal ions were negligible. Upon the addition of only 1.0 equivalent of Cu^2+^ to the 1:10 mixture of RuMAZO and other metal ions, a significant luminescence enhancement was observed, indicating that the existence of those metal ions in testing samples did not interfere copper(II) detection and imaging. Different copper salts (CuSO_4_, CuCl_2_, Cu(NO_3_)_2_ and Cu(OAc)_2_) were also tested, not much affection can be observed on the response of RuMAZO to Cu^2+^ ions with the presence of different counter anions (Fig. S6).

RuMAZO was observed to exhibit good photostability under the irradiation of 500 W iodine-tungsten lamp for 2 h (Fig. S7), this is beneficial for long-time luminescence tracking. In addition, the influence of pH on the luminescence of RuMAZO and RuTAZO was examined by luminescence titration under different pH value. As shown in Fig. S8, no obvious signal changes were observed over the pH range of 2–13, confirming that the luminescence of RuMAZO and RuTAZO was independent of pH and expected to work well under physiological conditions.

Amino acids were also examined as potential interfering factors for bioimaging applications of the probe. The result demonstrated that the presence of amino acids had no interference with the sensitive detection of Cu^2+^ by RuMAZO (Fig. S9). All these proved that RuMAZO is appropriate for biological Cu^2+^ sensing and imaging.

In summary, a fully water-soluble ruthenium(II) complex (RuMAZO) with o-(phenylazo)aniline group as reactive site has been developed as a turn on copper(II) luminescence sensor. Under a physiological environment (20 mM HEPES buffer solution, pH 7.4; 37°C), non-emissive RuMAZO can be efficiently transformed into high luminescent RuTAZO by an oxidative cyclization reaction with Cu^2+^ within 30 min, which can be triggered by nM-level (4.42 nM) concentration of Cu^2+^ with excellent selectivity. Moreover, the probe has been employed to image Cu^2+^ in live pea aphids with a turn-on luminescence signal.

## Methods

All solvents and chemical reagents employed for synthesis were analytical grade and purchased from commercial suppliers. The solutions of EDTA and metal ions were prepared from either their chloride or their nitrate salts. Deionized water was used as solvent. HEPES buffered aqueous solution (20 mM, pH = 7.4) was prepared in double-distilled water. Pea aphids, four-day-old, were obtained from Key Laboratory of Applied Entomology of Northwest A&F University. ^1^H NMR and ^13^C NMR spectra were recorded on a Bruker 500 AVANCE III spectrometer with chemical shifts reported in ppm at room temperature. Mass spectra were obtained with Thermo Fisher LCQ Fleet mass spectrometer (USA) and a LC/Q-Tof MS spectrometry (USA). The pH of the testing systems was determined by a PHS-3C pH Meter (China). Absorption spectra were collected by using a Shimadzu 1750 UV-visible spectrometer (Japan). Emission spectra were measured with a Shimadzu RF-5301 fluorescence spectrometer (Japan). Microinjection experiments were carried out by using a Drummond Nanoject II™ Auto-Nanoliter Injector (USA). Images of pea aphids were performed on an Olympus FV1000 confocal microscope (Japan).

Compound **1** was prepared according to the literature^44^. The MAZO ligand was prepared through the coupling reaction of compound **1** with phenyl diazonium salt^45^. The ruthenium(II) complex was obtained in a satisfactory yield (89%) through direct reaction of MAZO with the appropriate molar ratios of *cis*-[Ru(phen)_2_Cl_2_] in ethanol^46^.

### MAZO

Aniline (186 mg, 2.0 mmol) was dissolved in 2 mL concentrated hydrochloric acid, then 8 mL cold solution of NaNO_2_ (138 mg, 2.0 mmol) was added. The mixture was stirring under 0°C for 1 h. Then it was added into 36 mL 5-amino-1,10-phenanthroline (400 mg, 2.05 mmol) acetate buffer (3.0 g sodium acetate and 6 mL acetate) in dropwise in 30 minutes. After the addition was complete, the mixture was stirred for 24 h. Then mixture was filtered and the filtrate was suspended in 50 mL 3% ammonia. Stirred overnight and then filtered, washed with pure water, recrystallized in absolute ethanol. The yield was 0.548 g, 89.4%. ^1^H NMR (500 MHz, DMSO-*d*_6_): δ (ppm) 9.16 (dd, *J* = 3.1, 1.6 Hz, 1H), 9.15 (s, 1H), 9.07 (dd, *J* = 8.4, 1.5 Hz, 1H), 8.84 (dd, *J* = 4.2, 1.7 Hz, 1H), 7.99 (dd, *J* = 8.3, 1.0 Hz, 2H), 7.85 (dd, *J* = 8.4, 4.3 Hz, 1H), 7.70 (dd, *J* = 8.4, 4.2 Hz, 1H), 7.59 (t, *J* = 8.0 Hz, 2H), 7.46 (t, *J* = 7.3 Hz, 1H). ^13^C NMR (125 MHz, DMSO-*d*_6_): δ (ppm) 153.37, 152.28, 147.91, 146.74, 140.94, 137.73, 133.38, 130.67, 129.93, 129.88, 129.67, 124.43, 123.48, 122.33, 122.00, 121.68. ESI-MS: 300.16, [M + H]^+^; 322.11, [M + Na]^+^.

### RuMAZO

*cis*-[Ru(phen)_2_Cl_2_]·2H_2_O (0.284 g, 0.5 mmol) and MAZO (0.150 g, 0.5 mmol) were dissolved in 50 mL anhydrous ethanol, then the mixture was refluxed for 10 h under nitrogen. The mixture was concentrated to 2 mL; the residue was dropped to NH_4_PF_6_ solution and stirred for 30 minutes. Orange precipitate was filtered and washed with cold water. The crude product was purified by column chromatography on alumina with CH_2_Cl_2_/CH_3_CH_2_OH (100:1, v/v) as the eluent. Yield: 0.525 g, 89%. ^1^H NMR (500 MHz, Acetone-*d*_6_): δ (ppm) 9.38 (dd, *J* = 8.6, 1H), 9.22 (dd, *J* = 8.5, 1H), 8.88–8.75 (m, 4H), 8.57 (dd, *J* = 5.3, 1H), 8.53 (dd, *J* = 5.3, 1H), 8.47 (dd, *J* = 5.3, 1H), 8.43 (dt, *J* = 4.0, 4H), 8.41 (dd, *J* = 5.2, 1H), 8.38 (dd, *J* = 5.2, 1H), 8.12–8.04 (m, 3H), 7.92–7.85 (m, 2H), 7.84–7.78 (m, 3H), 7.68 (dt, *J* = 20.3, 1H), 7.61 (dd, *J* = 10.5, 2H), 7.52 (t, *J* = 7.3, 1H). ^13^C NMR (125 MHz, Acetone-*d*_6_): δ (ppm) 154.96, 154.09, 154.01, 153.86, 153.75, 150.68, 149.62, 148.86, 148.82, 148.78, 148.76, 143.34, 138.77, 137.80, 134.31, 133.86, 131.94, 131.89, 130.66, 131.11, 130.29, 129.05, 127.38, 127.12, 127.06, 127.03, 126.30, 122.82. HR-MS: 906.1203, [M-PF_6_^−^]^+^; 380.5777, [M-2PF_6_^-^]^2+^.

RuMAZO was then converted to the chloride salt by dissolving in a minimum amount of acetone, and then dropped to a saturated solution of tetrabutylammonium chloride in acetone, stirred for 15 minutes. The chloride salt was filtered, washed with acetone, and dried under vacuum. Yield: 0.340 g, 92%. ESI-MS: 796.00, [M-Cl^−^]^+^; 380.97, [M-2Cl^−^]^2+^.

### RuTAZO

RuMAZO (53 mg, 0.05 mmol) was dissolved in 10 mL ethanol and water (2/3, V/V), then CuSO_4_·5H_2_O (25 mg, 0.1 mmol) was added to the mixture. It was refluxed for 1 h. Then the mixture was concentrated and purified by chromatography to get red orange solid 50 mg, 94.7%. ^1^H NMR (500 MHz, Acetone-*d*_6_): δ (ppm) 9.17 (dd, *J* = 8.2, 1.1 Hz, 2H), 8.91–8.76 (m, 4H), 8.62–8.54 (m, 2H), 8.46 (ddd, *J* = 7.7, 6.0, 1.6 Hz, 4H), 8.44 (s, 4H), 8.40 (dd, *J* = 5.2, 1.1 Hz, 2H), 7.91 (dd, *J* = 8.2, 5.4 Hz, 2H), 7.86 (dd, *J* = 8.3, 5.3 Hz, 2H), 7.82 (dd, *J* = 8.3, 5.2 Hz, 2H), 7.76 (t, *J* = 8.0 Hz, 2H), 7.66 (t, *J* = 7.4 Hz, 1H). ^13^C NMR (125 MHz, Acetone-*d*_6_): δ 153.46, 153.20, 153.08, 150.10, 148.01, 147.92, 140.61, 139.83, 137.16, 137.15, 131.80, 131.17, 130.05, 129.80, 128.26, 127.34, 126.30, 126.27, 124.55, 124.55, 120.08. HR-MS: 904.1257, [M-PF_6_^−^]^+^; 379.5697, [M-2PF_6_^-^]^2+^.

## Author Contributions

S.G.S. supervised and interpreted the research. Y.F.Z. and Z.L.L. performed the measurements and wrote the manuscript. K.Y. performed the cells imaging and Y.Z. performed pea aphids imaging. Y.Q.X., H.J.L., C.X.W. and A.P.L. helped with interpreted data and wrote the manuscript. All authors discussed the results and commented on the manuscript.

## Supplementary Material

Supplementary Information Supplementary Information
