# Supplementary material for: A ruthenium(II) complex as turn-on Cu(II) luminescent sensor based on oxidative cyclization mechanism and its application in vivo
Source: Sci Rep. 2015 Feb 2;5:8172. doi: 10.1038/srep08172 (PMC4313082; doi:10.1038/srep08172)
Supplement: Supplementary Information [file srep08172-s1.doc]

A ruthenium(II) complex as a turn-on Cu(II) luminescent sensor based on oxidative cyclization mechanism and its application in vivo

Yunfei Zhang^1*^, Zonglun Liu^1*^, Kui Yang^1^, Yi Zhang^2^, Yongqian Xu^1^, Hongjuan Li^1^, Chaoxia Wang^3^, Aiping Lu^4^ & Shiguo Sun^1^

**Affiliations**

^1^College of Science, Northwest A&F University, Yangling, Shaanxi, 712100, China.

^2^College of Plant Protection, Northwest A&F University, Yangling, Shaanxi, China.

^3^Key Laboratory of Eco-Textile, Ministry of Education, School of Textile and Clothing, Jiangnan University, 1800 Lihu Avenue Wuxi, 214122, China.

^4^School of Chinese Medicine, Hong Kong Baptist University, Kowloon Tong, Hong Kong, China.

*These authors contributed equally to this work.

**Corresponding author**

Correspondence to: Shiguo Sun, sunsg@nwsuaf.edu.cn

**Table of contents**

1. Determination of quantum yield

2. The calculation of LOD

3. Kinetics of luminescence enhancement profile

4. Fluorescence spectra of the probe

5. ^1^H NMR ^13^C NMR and MS spectra

1. **Determination of quantum yield**

The quantum yield of compound RuTAZO was determined according to the literature^1,2^.


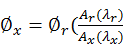
)(
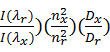


Where Φ is quantum yield; the subscripts x and r refer to the unknown and the standard respectively; λ is the excitation wavelength; A is absorbance at the excitation wavelength λ; I is the intensity of the exciting light at wavelength λ; n is the refractive index of the solution; D is the integrated area under the corrected emission spectrum. [Ru(bpy)_3_]Cl_2_ with the quantum yield of 0.028 in aerated water was chosen as the standard.

Table 1. Spectroscopic data for RuTAZO

| Compounds | λ_abs_(nm) | ε(10^4^ M^-1^cm^-1^) | λ _em_(nm) | Φ_f_ |
| --- | --- | --- | --- | --- |
| [Ru(bpy)_3_]Cl_2_ | 452 | -- | 597 | 0.028 |
| RuMAZO | 452 | 2.24 | 580 | N.D. |
| RuTAZO | 452 | 3.27 | 599 | 0.047 |

1. **The calculation of LOD**

The detection limit was calculated based on the fluorescence titration. Increasing amounts of Cu^2+^ were added to the probe (10 μM) in HEPES buffer. Representation of fluorescence at the appropriate wavelength vs. concentration of Cu^2+^ allowed the limit of detection to be calculated^3-5^.


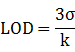


Where σ is the standard deviation of the blank solution measured by 10 times; k is the slope of the calibration curve.

1. **Kinetics of luminescence enhancement profile**

**Fig. S1** Time-dependent luminescence intensity of RuMAZO (10 μM) at 599 nm treated with various concentrations of Cu^2+^ in HEPES buffer (20 mM, pH 7.4, 37˚C) with λ_ex_ = 465 nm.

1. **Fluorescence spectra of the probe**

**Fig. S2** UV-Vis spectral changes of RuMAZO (10 μM) upon the addition of various concentrations of Cu^2+^ (0-30 μM) in a HEPES buffer solution (20 mM, pH 7.4, 37˚C).

**Fig. S3** Absorption (left) and emission (right) spectra of RuMAZO, RuMAZO + Cu^2+^ and RuTAZO (The concentration of RuMAZO, RuMAZO + Cu^2+^ and RuTAZO is 10 μM, the concentration of Cu^2+^ is 20 μM) in HEPES (20 mM, pH 7.4) at 37˚C.

**Fig. S4** The emission spectra of RuMAZO (10 μM; dotted line), RuMAZO (10 μM) + Cu^2+^ (10 μM; dashed line), and RuMAZO (10 μM) + Cu^2+^(10 μM) + EDTA (100 μM, 1000 μM; solid line) in HEPES buffer solution (20 mM, pH 7.4). λ_ex_ = 465 nm, λ_em_ = 599 nm.


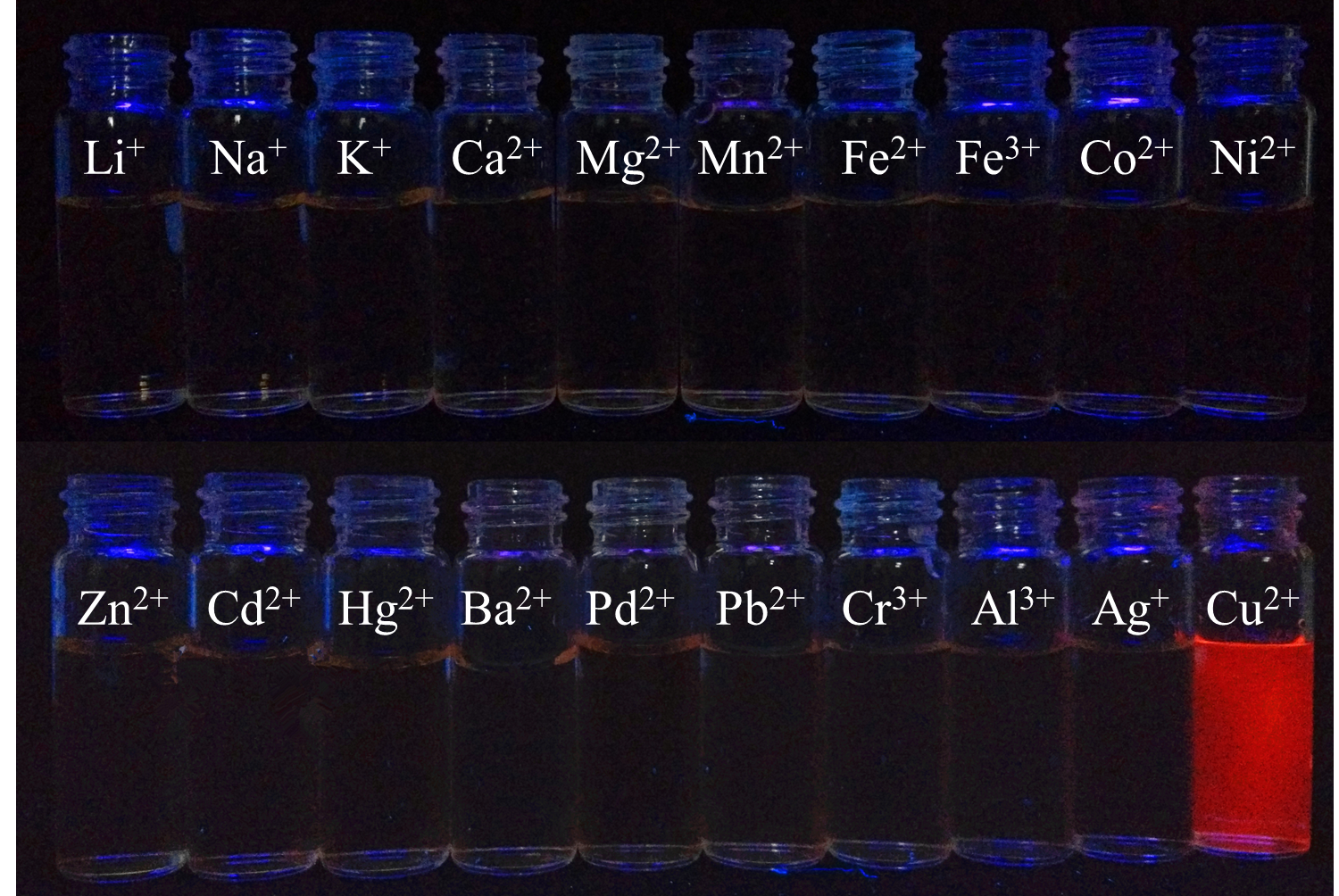


**Fig. S5** Luminescence changes photograph of RuMAZO (10 µM) upon the addition of various metal ions (100 µM; Li^+^, Na^+^, K^+^, Ca^2+^, Mg^2+^, Mn^2+^, Fe^2+^, Fe^3+^, Co^2+^, Ni^2+^, Zn^2+^, Cd^2+^, Hg^2+^, Ba^2+^, Pd^2+^, Pb^2+^, Cr^3+^, Al^3+^, Ag^+^) and 10 µM Cu^2+^.

**Fig. S6** The emission spectra of RuMAZO (10 μM), RuMAZO (10 μM) + Cu(OAc)_2_ (20 μM), RuMAZO (10 μM) + CuCl_2_(20 μM), RuMAZO (10 μM) + Cu(NO_3_)_2_ (20 μM) and RuMAZO (10 μM) + CuSO_4_(20 μM) in HEPES buffer solution (20 mM, pH 7.4) at 37˚C, incubated for 30 min. λ_ex_ = 465 nm, λ_em_ = 599 nm.

**Fig. S7** Time dependent luminescence change of RuMAZO and RuTAZO irradiated under 500 W iodine-tungsten lamp (λ_ex_ = 465 nm, λ_em_ = 599 nm for RuMAZO and RuTAZO).

**Fig. S8** The luminescence intensity of RuMAZO (10 μM) and RuMTAZO (10 μM) as a function of pH (2-13).

**Fig. S9** Luminescence changes of RuMAZO (10 µM) upon the addition of various amino acids (20 µM) and 20 µM CuSO_4_ in HEPES buffer solution (20 mM, pH 7.4) at 37˚C. Left-hand bars represent the luminescence responses toward amino acids (blank, Ala, Leu, Thr, Lys, Hcy, His, Cys, Glu, Met, Pro, Trp, GSH); right-hand bars represent the subsequent addition of 20 µM CuSO_4_ to the aforementioned solutions.

**Fig. S10** Percentage of HeLa cell viability remaining after cell treatment with RuMAZO, Cu^2+^, and RuMAZO in the presence of Cu^2+^ (the untreated cells were considered to have 100% survival).


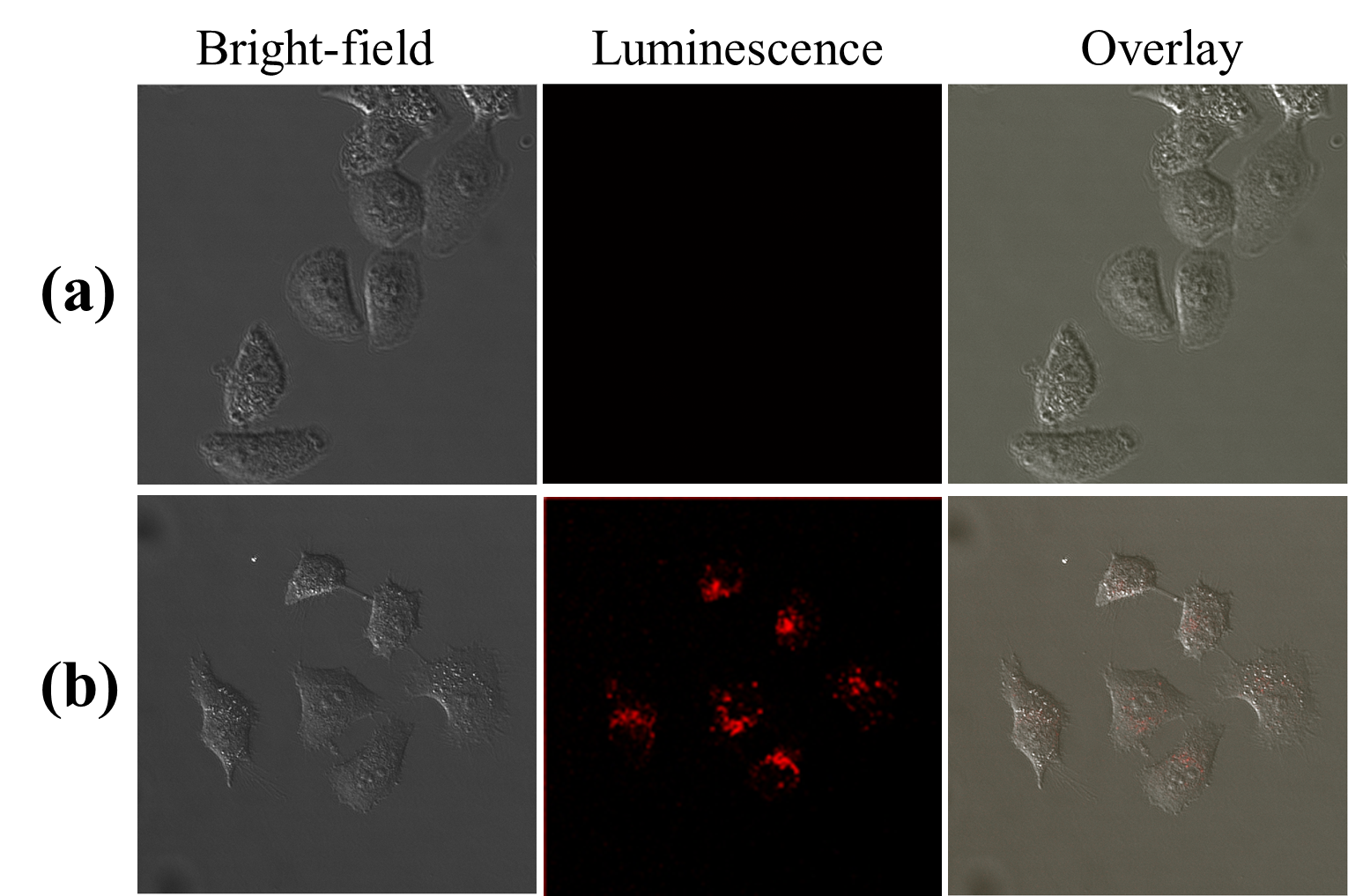


**Fig. S11** Luminescence imaging of HeLa cells incubated with RuMAZO (10 μM) for 2 h at 37˚C. (a) HeLa cells incubated with RuMAZO. (b) HeLa cells incubated with RuMAZO, then further incubated with the exogenous Cu source treatment (addition of 20 μM CuCl_2_ and 100 μM PDTC).

1. **^1^H NMR and ^13^C NMR spectra**

**Fig. S12** ^1^H NMR spectrum of ligand MAZO

**Fig. S13** ^13^C NMR spectrum of ligand MAZO

**Fig. S14** MS spectrum of ligand MAZO

**Fig. S15** ^1^H NMR spectrum of RuMAZO

**Fig. S16** ^13^C NMR spectrum of compound RuMAZO

**Fig. S17** HRMS spectrum of RuMAZO

**Fig. S18** ESI-MS spectrum of RuMAZO

**Fig. S19** ^1^H NMR spectrum of RuTAZO

**Fig. S20** ^13^C NMR spectrum of compound RuTAZO

**Fig. S21** HR-MS spectrum of RuTAZO

**References:**

1. Crosby, G.A. & Demas, J.N. Measurement of photoluminescence quantum yields. Review. *J. Phys. Chem.***75**, 991-1024 (1971).

2. Nakamaru, K. Synthesis, Luminescence Quantum Yields, and Lifetimes of Trischelated Ruthenium Mixed-ligand Complexes Including 3,3'-Dimethyl-2,2'-bipyridyl. *Bull. Chem. Soc. Jpn* **55** 2697-2705 (1982).

3. Thomsen,V. Schatzlein, D. & Mercuro, D. Limits of Detection in Spectroscopy. *Spectroscopy* **12**, 112-114 (2003).

4. Hakonen, A. Plasmon Enhancement and Surface Wave Quenching for Phase Ratiometry in Coextraction-Based Fluorosensors. *Anal. Chem.* **11**, 4555-4559 (2009).

5. Goswami, S. et al. CHEF induced highly selective and sensitive turn-on fluorogenic and colorimetric sensor for Fe^3+^. *Dalton Trans.* **42**, 15113-15119 (2013).
